# Selected static foot assessments do not predict medial longitudinal arch motion during running

**DOI:** 10.1186/s13047-015-0113-6

**Published:** 2015-10-12

**Authors:** Ben Langley, Mary Cramp, Stewart C. Morrison

**Affiliations:** Sport and Physical Activity, Edge Hill University, St Helens Road, Ormskirk, Lancashire L39 4QP UK; Human Motor Performance Group, School of Health, Sport and Bioscience, University of East London, London, UK; Centre of Health and Clinical Research, University of the West of England, Bristol, UK; School of Health Sciences, University of Brighton, Bristol, UK

**Keywords:** Foot, Running, Kinematics, Medial longitudinal arch, Static foot assessment

## Abstract

**Background:**

Static assessments of the foot are commonly advocated within the running community to classify the foot with a view to recommending the appropriate type of running shoe. The aim of this work was to determine whether selected static foot assessment could predict medial longitudinal arch (MLA) motion during running.

**Methods:**

Fifteen physically active males (27 ± 5 years, 1.77 ± 0.04 m, 80 ± 10 kg) participated in the study. Foot Posture Index (FPI-6), MLA angle and rearfoot angle were measured in a relaxed standing position. MLA motion was calculated using the position of retro-reflective markers tracked by a VICON motion analysis system, while participants ran barefoot on a treadmill at a self-selected pace (2.8 ± 0.5 m.s^−1^). Bivariate linear regression was used to determine whether the static measures predicted MLA deformation and MLA angles at initial contact, midsupport and toe off.

**Results:**

All three foot classification measures were significant predictors of MLA angle at initial contact, midsupport and toe off (*p* < .05) explaining 41–90 % of the variance. None of the static foot classification measures were significant predictors of MLA deformation during the stance phase of running.

**Conclusion:**

Selected static foot measures did not predict dynamic MLA deformation during running. Given that MLA deformation has theoretically been linked to running injuries, the clinical relevance of predicting MLA angle at discrete time points during the stance phase of running is questioned. These findings also question the validity of the selected static foot classification measures when looking to characterise the foot during running. This indicates that alternative means of assessing the foot to inform footwear selection are required.

**Electronic supplementary material:**

The online version of this article (doi:10.1186/s13047-015-0113-6) contains supplementary material, which is available to authorized users.

## Background

Recent systematic reviews [1, 2] have established that static foot posture is a risk factor for the development of overuse injuries of the lower extremity. The link between static foot posture and injury risk has long been held within the running community [3–5] and is a common view among recreational runners [6]. Injury risk associated with different foot types [1, 2, 7–9] has informed the development of running shoes for runners with different foot types [10]. As such static assessments of the structural alignment and/or characteristics of the foot are commonly advocated by footwear manufacturers, retailers and publications within the running community, for selection of the most appropriate type of running shoe [11–15].

Static foot classification techniques are underpinned by the premise that structure dictates function, and therefore the structural alignment or position of the foot, or aspects of the foot, can be used to predict dynamic foot motion [16, 17]. However, the association between static foot posture and dynamic foot function is poorly understood [1]. This is particularly true when dynamic foot function is assessed during running. Few studies [18–21] have explored the relationship between static foot assessments and dynamic foot motion during running, with conflicting outcomes. In an early study, Nachbauer and Nigg [21] explored the relationship between static MLA height and MLA deformation during running. These authors reported no significant relationship between static and dynamic measures. In contrast, more recent work by McPoil and Cornwall [19] reported that alterations in static medial longitudinal arch angle (MLAA) explained 85 % of the variance in MLAA at midsupport (MS) during running. Further studies [18, 20] demonstrated that static MLA height accounted for between 25 and 35 % of the variance in maximum rearfoot eversion. Additionally, Lee and Hertel [18] revealed that 74 % of the variance in maximum rearfoot eversion during running was accounted for by navicular drop.

Disparity in the current literature may be explained by the different measures used to classify the static foot and to quantify foot motion. Static foot assessments thus far have centred on measures of the MLA (e.g., MLAA, MLA height and navicular drop). As such, the literature to date has only explored how sagittal plane deviations in standing foot posture relate to dynamic foot motion during running. Alternative static classification tools such as the rearfoot angle (RFA) and Foot Posture Index (FPI-6) provide frontal and multi planar assessments respectively and have previously been related to overuse injuries [22, 23]. Furthermore, these classification tools have also been shown to explain between 21 and 85 % of the variance in variables associated with dynamic foot motion during walking [16, 24–27]. These findings suggest that the RFA and FPI-6 may be appropriate measures for predicting dynamic foot motion. However, the relationship between these measures and dynamic foot motion during running has yet to be explored. The aim of this study was to determine whether the aforementioned static foot classification measures could predict MLA motion during running. Due to the exploratory nature of this investigation an *a priori* hypothesis was not proposed.

## Methods

### Participants

Fifteen active males (27 ± 5 years, 1.77 ± 0.04 m, 80 ± 10 kg) participated in the study and were recruited from the University of East London and local sports clubs. Health screen and physical activity questionnaires were completed by all participants prior to data collection. All participants were free from musculoskeletal injury or deformity, cardiovascular problems or illness at the time of testing. On average participants reported exercising three to four times per week which included running two to three times per week. Written informed consent was obtained from each participant prior to testing. Ethical approval was granted for this study by the University of East London Research Ethics Committee.

### Foot classification measures

A single examiner (BL) conducted all foot classification measures. From the literature, four potential foot classification measures were identified; FPI-6, MLAA, navicular drop and RFA. Pragmatic decisions about the inclusion of each measure were made based upon the intra-rater reliability of each foot classification measure determined during pilot testing. All measures demonstrated at least substantial levels of reliability (*ICC*_(3, 1)_ > .69), except for the navicular drop measure which was fair (*Kw* = .4). As such the FPI-6, MLAA and RFA were used to classify the foot within this study.

**Fig. 1:**
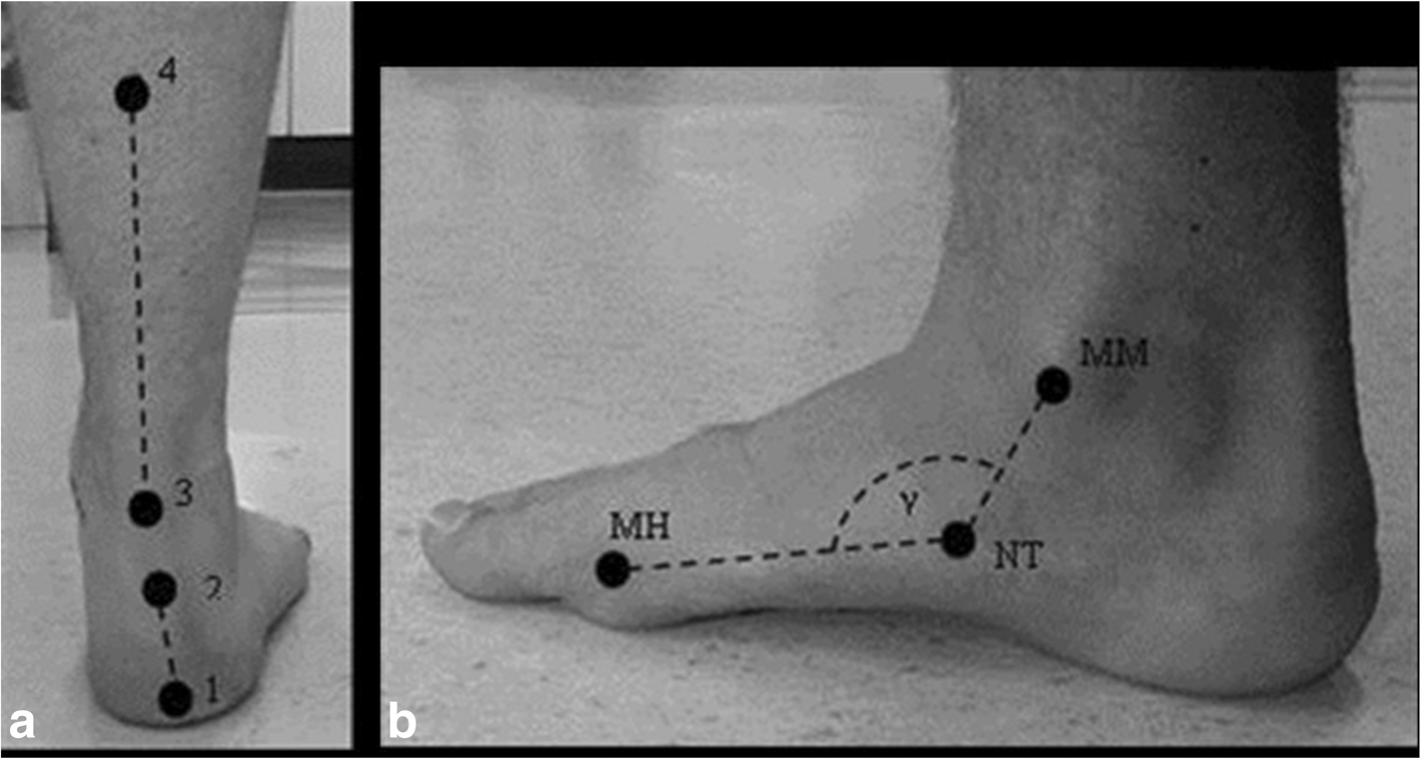
**a** Marker locations for RFA calculation. **b** Anatomical landmarks used to calculate the MLAA. **a** Marker 1 = base of calcaneus, marker 2 = Achilles tendon attachment, marker 3 = centre of Achilles tendon at the height of the medial malleous and marker 4 = centre of the posterior aspect of the shank 15 cm above marker 3. **b** MM = medial malleolus, NT = navicular tuberosity, MH = first metatarsal head and γ = MLAA

The right foot was assessed for each participant. For all foot classification measures, participants were asked to assume a relaxed standing position in double limb support, looking straight ahead with their arms by their sides. The foot classification measures were conducted in the following order; FPI-6, MLAA and RFA. The FPI-6 was conducted following a standard protocol [28]. Talar head congruency, lateral malleoli curvature, calcaneal inversion/eversion, talonavicular bulging, MLA congruency and forefoot to rearfoot abduction/adduction were assessed. Each component was scored on a scale ranging from −2 to +2 and the cumulative score used to define foot posture. The RFA was calculated from the reconstructed positions of four retro-reflective markers placed to indicate the central vertical lines of the posterior shank and rearfoot (Fig. 1a) and tracked by a VICON motion analysis system (VICON Motion Systems Ltd., Oxford, England). MLAA was calculated from the position of three retro-reflective markers tracked by the VICON motion analysis system. The markers were attached to the medial malleolus, navicular tuberosity and the first metatarsal head (Fig. 1b). Cut off points for each foot classification measure are shown in Table 1.

**Table 1:** Foot classification cut off points for the FPI-6, RFA and MLAA

	Pronated	Neutral	Supinated
FPI-6	>5	0 to 5	<0
RFA	≥3°valgus	2° valgus to 2° varus	≥3° varus
MLAA	<130°	130° to 150°	>150°

### Dynamic assessment

Kinematic data were collected using an eight camera VICON motion analysis system, operating at 200Hz. Participants ran barefoot on a Jaeger LE 300 C treadmill (Erich Jaeger GmBH & Co, Wuerzburg, Germany), at a self-selected pace (2.8 ± 0.5 m.s^−1^). Prior to data collection, participants undertook a 10 min familiarization period on the treadmill to reduce kinematic differences between overground and treadmill locomotor patterns [29, 30]. Data was collected continuously for the final 30 s of 3 min long dynamic trials.

Dynamic MLA motion was calculated within this study from the position of 14 mm retro-reflective markers tracked using the VICON system. This study was undertaken as part of a larger research project and markers were attached in accordance with standard lower limb [31] and foot [32] models. A static trial was recorded prior to dynamic trials. This enabled the relevant segment fixed reference frames to be calculated and the position of anatomical markers to be reconstructed in relation to the triad marker clusters for the foot model [32]. Upon completion of the static trial, all anatomical markers were removed and participants’ height, ankle width and mass were measured using a Seca 213 portable stadiometer (Seca, Chino, CA, USA), Holtain Ltd callipers (Holtain Ltd., Crymych, Wales) and Seca 761 Class IIII scales (Seca, Chino, CA, USA). To calculate the MLAA throughout dynamic trials, the calibrated anatomical system technique (CAST) [33] was used to reconstruct the position of anatomical markers located on the first metatarsal and navicular tuberosity from the position of triad marker clusters located on the navicular tuberosity and the midshaft of the first metatarsal head. The position of these markers and the lateral malleoli marker were used to calculate dynamic MLAAs. Prior to the dynamic MLAA calculation, the position of the lateral malleoli marker was projected medially using the ankle width, to give an indication of the medial malleoli position. To account for differences in the medial and lateral malleoli position, that would result in differences in MLAA recorded within the static and dynamic conditions, an angular offset was calculated for each participant. The angular offset was calculated as the difference between a participants static MLAA within the foot classification trial and the MLAA calculated using the medial projection of the lateral malleoli marker during the static trial prior to dynamic capture. This offset value was then applied to the MLAA calculated at each time point during dynamic trials. These calculations were undertaken using custom written MatLab script (MathWorks, Natick, Ma, USA).

Dynamic MLAA were averaged over five consecutive gait cycles for each participant and normalized to 100 % stance phase duration. Gaps, of up to five frames, in marker trajectories were filled using the in-built spline fill function within VICON Nexus 1.7.1 (Vicon Motion Systems Ltd., Oxford, England). Reconstructed marker positions were filtered using a 20Hz Butterworth filter within Matlab. Gait cycle parameters were identified from the kinematic data. The change in vertical velocity of the distal heel marker from negative to positive was used to identify initial contract (IC) and peak knee extension identified toe off (TO) [34]. MLAA at IC, MS (50 % stance phase) and TO of the gait cycle were extracted, as was MLA deformation. MLA deformation was calculated as the difference between minimal MLAA and MLAA at IC.

### Statistical analysis

Statistical testing was undertaken using SPSS 20 (IBM, Armonk, NY, USA). Descriptive statistics for each foot classification measure were calculated. Prior to statistical analysis FPI-6 scores were transformed into logit values through Rasch modelling to convert the ordinal scores into interval measures for parametric statistical analysis [35]. The sampling distribution of all regression pairings were checked for normal distribution using Shapiro-Wilk test. All data was normally distributed. Bivariate linear regression was used to determine the extent to which static foot classification measures predicted stance phase MLA deformation and MLAA at IC, MS and TO. The coefficient of determination was used to determine the extent to which differences in static foot classification scores explained the variance in MLA motion. The level of significance was *p* < .05.

## Results

Tables 2 and 3 contain descriptive statistics for foot classification measures and dynamic MLA motion respectively. All three foot classification measures were significant predictors of MLAA at IC, MS and TO during the stance phase of running (*r* = .64 to .95; *p* < .05) (Table 4). The FPI-6 had a moderate negative relationship with MLAA at IC and strong negative relationship at MS and TO during running. The static MLAA had a strong positive relationship with MLA motion at IC, MS and TO. Moderate positive relationships were reported between the static RFA and dynamic MLAA at IC and TO. The static RFA had a strong positive relationship with dynamic MLAA at MS. None of the static foot classification measures were significant (*p* > .05) predictors of MLA deformation throughout the stance phase of running gait.

**Table 2:** Descriptive statistics for the FPI-6, MLAA and RFA

	Mean (SD)	Minimum	Maximum
FPI-6^a^	4 (4)	−4	12
MLAA	132° (13°)	108°	151°
RFA	4° valgus (5°)	17° valgus	3° varus

^a^Raw data (non-transformed scores)

**Table 3:** MLAA at IC, MS, TO and MLA deformation during the stance phase of running (Mean (SD))

	IC	MS	TO	Deformation
MLAA	135° (12°)	128° (12°)	139° (14°)	8° (4°)

**Table 4:** Coefficient of determination (*r* ^*2*^) for static foot classification measures and aspects of dynamic MLA motion throughout the stance phase of the running gait cycle

	Dynamic MLA motion			
	IC	MS	TO	Deformation
FPI-6	.46*	.58**	.53**	.13
MLAA	.90***	.86***	.85***	.03
RFA	.46*	.58**	.41*	<.01

*p < .05 **p < .005 ***p < .001

## Discussion

The aim of this study was to determine whether selected static foot classification measures predicted MLA motion during running. Foot motion was measured using the MLAA as it is thought to give an indication as to how the foot functions throughout the gait cycle, taking into account rear- and mid-foot motion [36], with both discrete angles and deformation examined. The relationship between static foot classification measures and both MLAA at discrete time points and MLA deformation during the stance phase of running gait were explored in line with the previous literature [18–21]. Significant relationships were reported for all static foot classification measures and MLAA at IC, MS and TO. The findings demonstrated that static MLAA was the optimal means of predicting dynamic MLAA at IC, MS and TO. Variance in the static MLAA accounted for 90, 86 and 85 % of the differences in MLAA at IC, MS and TO respectively. Variation in FPI-6 scores accounted for 46, 58 and 53 % of the variance in MLAA at IC, MS and TO respectively. Static RFA accounted for 46, 58 and 41 % of the variation in MLA angles at IC, MS and TO respectively. No significant relationship was found between the static foot classification measures and MLA deformation during running.

The strong relationships (*r* ≥ .91) reported within this study between static MLAA and MLAA at discrete time points within the stance phase were expected on the basis of previous literature [19]. The strong relationships between these variables are liable to be a result of both variables measuring the same construct (the MLA). Large deviations in dynamic MLAAs, at discrete time points within the running gait cycle, from static MLAAs would not be expected. Analysis of the descriptive statistics supports this assumption with static and dynamic MLAAs at discrete time points having similar magnitude and variance (Tables 2 and 3). These findings suggest that participants with a static MLAA of around 130° will have dynamic MLAAs around 130°, which is likely to explain the high levels of association reported both within this study and the previous literature [19]. As such the levels of explained variance between each static foot classification measure, in relation to dynamic MLAA at discrete time points within the gait cycle (Table 4), are liable to be explained by differences in the parameters measures by each of the selected foot classification tools. This highlights that the selected static foot classification tools, which assess different parameters associated with the structural alignment of the foot, are not analogous in the manner in which they classify the foot.

While moderate to strong relationships (*r* > .67) have been reported between within this study between static foot classification measures and dynamic MLAA at discrete time points during the stance phase of running gait (Table 4), the usefulness of being able to predict these angles is questionable. The relationships between each static foot classification measure and dynamic MLAA at discrete time points provides only an indicator as to whether or not the MLAA is higher or lower at a given time point, as oppose to an indication as to the magnitude of MLA deformation. Although the link between foot type and running related injury is still not well understood, it is MLA deformation that has theoretically been associated with increased running related injury risk [7, 37]. For this reason the authors believe that the key findings within this study was the lack of a significant relationship between static foot classification measures and dynamic MLA deformation during running. The lack of a significant relationship between any of the selected static foot classification measures and MLA deformation during running reported within this study is consistent with the findings of Nachbauer and Nigg [21]. These findings highlight the limitations of static foot classification measures when looking to predict the magnitude of dynamic foot motion, and has implications within the running community; where static foot classification measures are commonly used to recommend running shoes [11–15]. This study therefore advocates moves away from static foot assessment, when looking to predict the magnitude of dynamic foot motion.

The findings from this study need to be interpreted in light of the limitations. The work was exploratory and as a result the sample size was small and there was limited representation of foot types, with no representation of the supinated foot type. The spread of foot classification scores, within this study, were clustered on the boundary between the pronated and neutral foot classification groups (Table 2). This factor may have increased the homogeneity within the sample population, reducing the variance in MLAA motion patterns between participants. The small sample size also restricted the current study to bivariate analysis and as such the ability of multivariate models to predict dynamic MLA motion was not explored. Additionally, the use of the lateral malleoli marker to estimate the position of the medial malleoli during dynamic trials may potentially be viewed as a limitation of the study. The lateral malleoli marker was used for the MLAA calculation out of necessity. Initially the MLA height to length ratio calculated within the Jenkyn and Nicol [32] foot model was identified as a measure of dynamic foot function. However, the height to length ratio was found to lack robustness (see Additional file 1). For two participants a marker was placed on the medial malleoli throughout the dynamic trials in order to compare MLAA calculated using the medial malleoli marker and the medial projection of the lateral malleoli marker. Comparisons of the dynamic MLA motion calculated by these two methods revealed mean differences of 3° between the two methods of calculating dynamic MLAAs. This therefore suggests that the method used to calculate MLAAs within this study accurately replicate those measured directly using a marker located on the medial malleoli. However, further comparisons of the MLAA calculation method used within this study and MLAA calculation using a medial malleoli marker are required to further determine the validity and accuracy of this approach.

## Conclusions

The three foot classification measures used in this study were significant predictors of the MLAA at IC, MS and TO. The static MLAA was the optimal means of predicting dynamic MLAA at discrete time points within the running gait cycle. However, the relevance of predicting MLAA at discrete time points is debatable. MLA deformation not angles at discrete time points has theoretically been linked to running related injuries. The key finding within this work was that the selected static foot classification measures did not predict dynamic MLA deformation. This finding questions the purpose of using static foot classification measures when looking to characterise the foot during running.

## Additional file

Additional file 1:
**Medial longitudinal arch calculation rationale and supporting data.** (DOCX 1365 kb)

## Abbreviations

MLA: Medial Longitudinal Arch
MLAA: Medial Longitudinal Arch Angle
RFA: Rearfoot Angle
FPI-6: Foot Posture Index
IC: Initial Contact
TO: Toe Off
MS: Midsupport
