# Supplementary material for: Selected static foot assessments do not predict medial longitudinal arch motion during running
Source: J Foot Ankle Res. 2015 Oct 12;8:56. doi: 10.1186/s13047-015-0113-6 (PMC4603633; doi:10.1186/s13047-015-0113-6)
Supplement: Additional file 1: — Medial longitudinal arch calculation rationale and supporting data. (DOCX 1365 kb) [file 13047_2015_113_MOESM1_ESM.docx]

Additional Material: Selected static foot assessments do not predict medial longitudinal arch motion during running

MLA motion is calculated as a ratio of MLA height to length in the Jenkyn and Nicol [32] foot model (Supplementary Figure 1A); with an increasing ratio representing a supinating foot and a decreasing ratio a pronating foot. MLA height is defined as the distance between the navicular tuberosity marker (NT) and the MLA length vector, which is defined as the distance from the medial aspect of the calcaneus (CAMT) and the first metatarsal head (1MH). The model assumes that NT is above the MLA length vector. However, within this study, we found that participants with low arch profiles violated this assumption as the navicular marker was below the vector (Supplementary Figure 1B). This reversed the calculated motion curve, with increasing ratios representing a pronating foot and decreasing ratios a supinating foot, resulting in disparity between MLA motion curves between participants. Additionally, if the navicular marker was close to the length vector there was the potential for the marker to cross the vector during the dynamic trials, again altering the calculated ratio. This, therefore, led to the conclusion that the measure was not robust and as such it was desirable to use an alternative measure of MLA motion. As such the MLAA was calculated as outlined within the methods using the position of the lateral malleoli.


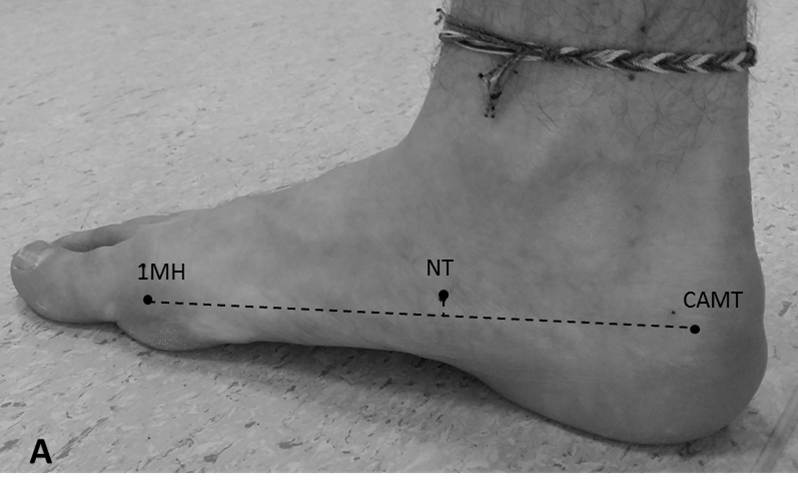

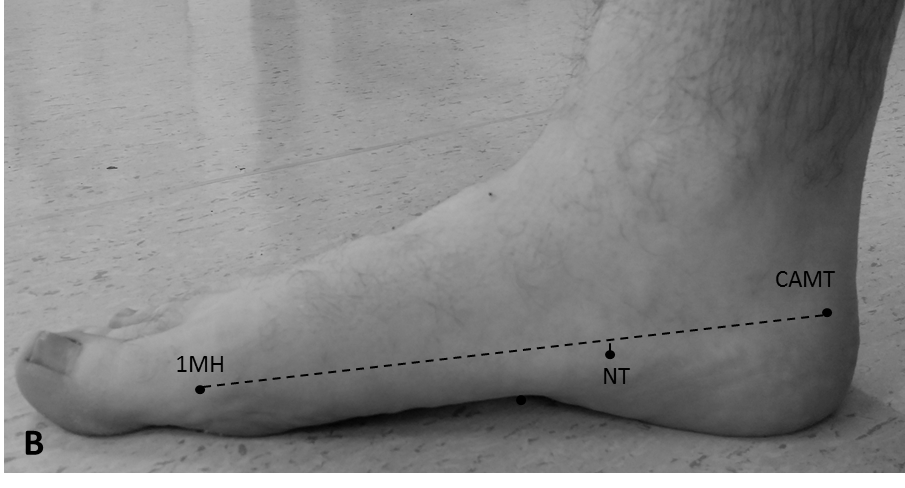


Supplementary Figure 1. Marker locations for the calculation of the MLA height to length ratio within the Jenkyn and Nicol (2007) foot model. A) Neutral foot with navicular marker above the MLA length vector. B) Pronated foot with navicular marker below the MLA length vector

Supplementary Figure 1. Marker locations for the calculation of the MLA height to length ratio within the Jenkyn and Nicol (2007) foot model. A) Neutral arch profile (neutral foot) with navicular marker above the MLA length vector. B) Low arch profile (pronated foot) with navicular marker below the MLA length vector

Supplementary Figure 2 shows the MLA motion curves, averaged over two participants who were retested, calculated from the position of markers attached to the lateral malleoli and also the medial malleoli. The difference between the two curves was calculated, at each time point during the stance phase, and the average calculated. Supplementary Figure 2 shows that there is a systematic upward shift in the MLA angle calculated using the lateral malleoli position. The mean difference between the two MLA curves was 3°. Supplementary Figure 3 further highlights the similarity between the two motion curves, each curve was demeaned and then plotted so that the motion patterns moved around 0°, the difference between the two calculation methods here was <1°. This information has led to the conclusion that we can have a high level of confidence in the dynamic motion patterns calculated within the work.

Supplementary Figure 2. MLAA motion patterns throughout the stance phase of barefoot running calculated using a medial projection of the lateral malleoli marker (dashed line) and the medial malleoli marker (solid line); averaged motion patterns over 5 trials for 2 participants

Supplementary Figure 3. Demeaned MLAA motion patterns throughout the stance phase of barefoot running calculated using a medial projection of the lateral malleoli marker (dashed line) and the medial malleoli marker (solid line); averaged motion patterns over 5 trials for 2 participants
